# Factorial validity, measurement and structure invariance of the Malay language decisional balance scale in exercise across gender

**DOI:** 10.1371/journal.pone.0230644

**Published:** 2020-03-18

**Authors:** Garry Kuan, Abdulwali Sabo, Sukanlaya Sawang, Yee Cheng Kueh

**Affiliations:** 1 Exercise and Sports Science, School of Health Sciences, Universiti Sains Malaysia, Kubang Kerian, Kelantan, Malaysia; 2 Department of Life Sciences, Brunel University London, Uxbridge, United Kingdom; 3 Unit of Biostatistics and Research Methodology, School of Medical Sciences, Universiti Sains Malaysia, Kubang Kerian, Kelantan, Malaysia; 4 International Centre for Transformational Entrepreneurship, Coventry University, Coventry, United Kingdom; Murcia University, SPAIN

## Abstract

**Background:**

This study examined the psychometric properties of the Malay version of the decisional balance (DB-M) for exercise (i.e. perceived benefits and perceived barriers) using a cross-sectional design. Also, this study assessed the measurement and structural invariance of the DB-M across gender.

**Methods:**

The study sample consisted of 750 students (female: 51.7%, male: 48.3%), with a mean age of 20.2 years (SD = 1.2). Decision balance (DB) scale was assessed with the 10-item DB-M. Standard forward-backward translation was performed to translate the English version of the DB into Malay version (DB-M).

**Results:**

The confirmatory factor analysis (CFA) results based on the hypothesised measurement model of two factors and ten items demonstrated adequate factor structure after the addition of some correlated item residuals (comparative fit index (CFI) = .979, Tucker and Lewis index (TLI) = .969, standardised root mean square residual (SRMR) = .037, root mean square error of approximation (RMSEA) = .047). The construct reliability and average variance extracted values were .850 and .839, and .542 and .538, for perceived benefits and perceived barriers, respectively. Meanwhile, the Cronbach’s alpha was .857 and .859, and the intraclass correlation coefficient for test-retest reliability was .979 and .960 for perceived benefits and perceived barriers respectively. The findings provided evidence for measurement invariance of DB-M for the male and female samples. The final CFA model fit the data well for both male sample (CFI = .975, TLI = .964, SRMR = .040, RMSEA = .052) and female sample (CFI = .965, TLI = .949, SRMR = .044, RMSEA = .058).

**Conclusions:**

The translated version of the DB-M was valid and reliable for assessing the level of perceived benefits and perceived barriers in exercise among university students in Malaysia.

## Introduction

It has been widely reported that participation in regular physical activity can significantly reduce the risk of hypertension, diabetes, cardiovascular diseases, obesity, overall fitness, mental wellness, and quality of life [1, 2]. Frequent physical activity is an essential determinant that enhances the human body system (e.g. musculoskeletal, cardiovascular, and metabolic system) for optimal functioning [3]. However, in 2009, physical inactivity was reported as the fourth leading risk factor for noncommunicable diseases, causing about three million premature deaths [4]. In Malaysia, the prevalence of physical inactivity was 39.7% and that the population spent most of their time (74% of the day) in sedentary activities, such as sleeping or lying down [5].

The Transtheoretical Model (TTM) is among the most commonly used theories of the behaviour of change that has been successfully employed by researchers to describe a spectrum of health behaviours such as physical activity and health promotion [6]. The TTM is a collective psychological framework that aims to demonstrate deliberate health behaviour choice and sustenance as a process that transpires over time as a function of behavioural history and motivation [7]. This model illustrates how people move dynamically through stages of behavioural change [8]. Many studies have used the TTM or Stages of Behavioural Change (SBC) Model to develop interventions to promote physical activity [9, 10].

The TTM compromises the temporal dimension and the stages of change, and these were integrated into the process and principles of change of various testable theories. The TTM is applied to exercise behaviour to illustrate the capability and the extent of willingness and incremental shift over time, as well as its specification of transformation-related to and profile variations between phases [8]. The TTM, which depicts human readiness to change and advancement through a sequence of phases, involves five key constructs, i.e. stages of change, the process of change, decisional balance, self-efficacy and temptation [8]. This study focuses on the testing of the translated Malay version of the decisional balance components of the TTM.

The decisional balance construct reflects the struggle model, which is an essential process of making a decision associated with specific health practices [11]. Decisional balance signifies the perceived benefits (pros) and perceived barriers (cons) related to exercise behaviour. Perceived benefits for exercise refer to enhanced self-confidence, physical strength, and aerobic ability, while perceived barriers to exercise refer to physical distress, cost, and hesitation. Many researchers reported that the level of perceived benefits increases while the barriers decrease within the stages of behavioural change [8]. The decisional balance sheet (DBS) developed by Janis and Mann [11] is among the most assuring intervention for exercise domain [12]. The DBS is derived from the expectancy theory, which stated that the level of a person’s expectations of gains or failures is what determines his relative course of action. The more critical the information is viewed, before reaching a decision, the more effective the commitment to that decision and the more stable the adherence to the decision [11]. A study by Nigg and Courneya [12] confirmed that the DBS intervention was effective in maintaining current exercise behaviours, whereas, a decline in exercise behaviours was witnessed among the placebo groups over eight weeks.

The Decisional Balance (DB) scale was initially developed by Marcus and Rakowski [13], consisting of two subscales: (1) perceived benefits (pros, ten items) reflect the positive aspects of exercise and (2) the perceived barriers (cons, six items) reflect the negative aspects of exercise. The decisional balance scale was later revised and amended in a pilot study among 543 Canadian adults (18–65 years old). The modified decisional balance scale consisted of 10 items (five for pros and five for cons) assessed using the 5-point response options ranging from 1 (not at all important) to 5 (very much) [14]. Despite widely used measurement, the validity of DB scale for Asian population is still limited. Cultural variation may influence the individual's exercise experience and “thus, when researchers and practitioners employ Western-based assessments with Asian populations by directly translating them without an appropriate validation, the process can be challenging” [15]. In this study, we translated the decisional balance scale into Malay language (DB-M) and confirmed its psychometric properties based on the modified version with ten items.

Given that, several statistics suggest that adult physical inactivity is a significant public health concern in Malaysia [16]. Apart from that, many studies have shown that the patterns of physical activity behaviours practised in college are likely to be sustained for a long time [17, 18]. Generally, all college students are adults with various responsibilities [19]. As such, they are more likely to maintain physical activity behaviour that they acquire during their college years and throughout their adulthood; therefore, the habit determines long-term health [17]. According to Sullum, Clark and King [20], health educators attempting to overcome the risk of exercise relapse should focus on reducing the cons and then shift focus to improve pros. Therefore, in this study, we aimed to examine the psychometric properties of the Malay version of the decisional balance scale (DB-M) and confirm its measurement invariance across gender among university students in Malaysia.

## Methods

### Participants

A total of 800 questionnaires were administered, and 750 (93.8% response rate) with complete responses to all the items were returned to the researchers. Hence, the final data set was 750 (female: n = 388, 51.7%; male: n = 362, 48.3%) with no missing data. The participants had a mean age of 20.2 years (SD = 1.2) and they identified themselves as Malay (63.4%), Chinese (20.9%), Indian (9.7%), and others (6.0%), but they were all Malaysians with strong reading and speaking skills in the Malay language.

### Measures

The decisional balance scale was a self-report measure to evaluate the participants’ perception of the pros (or perceived benefits) and cons (or perceived barriers) of maintaining a prevailing behaviour or embracing a new behaviour [14]. Examples of pros items included, “Physical activity would help me reduce tension or manage stress” while examples of cons items included “Physical activity would take too much of my time” [14]. The internal consistency of the modified version was satisfactory, with Cronbach’s alpha of .79 for pros and .71 for cons [14].

### Questionnaire translation

The standard forward-backwards translation method was applied to translate the English version of the decisional balance scale into the Malay language [21]. First, a bilingual researcher translated the DB scale from English to Malay to retain the content meaning and not word for word translation. Second, a local Malay who was bilingual in Malay and English back translated the Malay version to English. Third, these two translated versions were reviewed by a panel of five bilingual experts from the field of health psychology, sport sciences, physical education, and sports psychology. The items were matched with its corresponding items in the original English version and were further assessed to ascertain whether they were culturally appropriate for Malaysian populations. Finally, a sample of 30 undergraduate students was invited to assess the items’ clarity, understanding, and comprehension. The students’ feedback was similar to one another and required no revisions.

### Data collection

This study was approved by the Human Research Ethics Committee of the Universiti Sains Malaysia. The study was a cross-sectional design and implemented between September and December 2018. The purpose of the study and method of data collection were explained to the students at the end of their lecture sessions by the researchers. The students who consented to participate were given the information sheets containing the study’s description before being asked to complete the questionnaire. Implied consent was considered to have been given when the participants voluntarily completed the DB-M questionnaire and returned it to the researchers. Time to complete the DB-M was at an average of 15 minutes. To report the test-retest reliability of the DB-M, 100 participants completed and returned the DB-M scale on day 14 after the first measurement.

### Statistical analysis

The Mardia multivariate normality of skew and kurtosis was initially conducted to examine the multivariate assumption of the data. The multivariate skew (p-value < .001) and kurtosis (p-value < .001) normality assumption was violated. Hence, the robust maximum likelihood estimator (MLR) was used because it provides robust estimates that are robust to non-normality violations [22, 23]. Many researchers have reported that MLR estimator can be used for ordinal CFA based models when the number of response levels for every item was equal to or greater than five [24, 25]. Also, a variable can be treated as a continuous measure when it is measured using a Likert scale with five or more response options [26]. Confirmatory factor analysis (CFA) along with measurement and structural invariances analyses were performed using Mplus version 8 to impose the hypothesised measurement structure on the data. The standardised factor loading of .40 or greater was used as a cut-off value to establish sufficient factor loading for all items of the DB-M scale. Items with factor loadings of less than .40 were considered to be uncertain items and were deleted with sufficient theoretical support [23].

Rather than applying the Chi-square goodness-of-fit (χ^2^), Normed chi-square (NC) = χ^2^/df was originated because of the model chi-square sensitivity to the sample size [27]. Nevertheless, there was no precise guideline regarding the maximum acceptable values of NC. Following chi-square goodness-of-fit sensitivity to sample size and given that normed chi-square has no use in global fit testing, it is therefore not reported in this study. In the present study, the Standardised Root Mean Square Residual (SRMR), Root Mean Square Error of Approximation (RMSEA), Tucker-Lewis Index (TLI) and Comparative Fit Index (CLI) for estimating the validity of the CFA model were the only fit indices presented. This is in line with the recommendation of Brown [28], which stated that "the most universally accepted global goodness of fit indices are the SRMR, RMSEA, TLI, and CLI.

The recommended fit indices for a sample size of more than 250 with less than 12 used observe variables are: RMSEA of less than .07, SRMR of less than .08, and CFI or TLI of .95 or higher [29]. The construct validity of the DB-M was further determined by computing the construct reliability (CR) and average variance extracted (AVE). CR was estimated using Raykov’s method [30] in Mplus 8.0. The cut-off values were equal to or greater than .60 for CR [31] and .50 for AVE [32]. Discriminant validity was established by assessing the correlation between the factors, and a correlation coefficient of less than .85 suggests the adequate discriminant ability of the model [33]. Additionally, model re-specification was done by adding the residual covariances of items within the same factor. These error covariances reveal the assumption that the two corresponding items share some unexplained variances in common that is not specified in the model [27]. The model re-specification was based on the modification index (MI) values and carried out after the researchers obtained sufficient theoretical support.

The hierarchical test of measurement invariance was performed across gender using the recommended guidelines for establishing measurement invariance of the DB-M model [22, 26, 34]. A step by step restrictive constraints was imposed on the model parameters, and the differences in the model fit indices were examined. To examine measurement invariance, first, the configured invariance model needs to be established to compare it with the fit indices of other invariance models. In the configural invariance model, no equality restrictions were imposed on the model parameters across gender. Second, the weak invariance or metric invariance model was established and examined. In the weak invariance model, equality restrictions were imposed on the model factor loadings across gender to guarantee the similarity of the measurement scale across gender and make accurate comparisons. Third, the strong invariance model was established and examined. In the strong invariance model, equality restrictions were imposed on the factor loadings, as well as item intercepts across gender to guarantee the scale factors similarities across gender. Finally, a strict invariance model was established and examined. In the strict invariance model, equality restrictions were imposed on the factor loadings, item intercepts, and residual variances to determine that the items’ variances of regression equations were invariant across gender.

The structural invariance of the model parameters was also investigated using the factor variance and factor covariance invariance, and the factor means invariance. The factor variance and factor covariance invariance were examined to ascertain the similarities of factors correlation across gender. In contrast, the factor means invariance was examined to ascertain the similarities of factors means differences across gender. In general, the structural invariance assessed the extent to which the male and female samples differ and not related to the measurement scale studied. For the present study, the recommended invariance cut-off values used were an absolute difference (Δ) of .01 or less for CFI (ΔCFI) and TLI (ΔTLI) and .015 for RMSEA (ΔRMSEA) [26, 35, 36].

In this study, the internal consistency of the DB-M was evaluated based on Cronbach’s alpha to compare it with the previous study’s Cronbach’s alpha [14, 37]. For test-retest reliability, the intraclass correlation coefficient (ICC) was computed using a subsample of the 100 participants’ scores. Cronbach’s alpha and ICC were computed using SPSS 24 and ICC values greater than .90 were considered to indicate excellent stability.

## Results

### Characteristics of the participants

The participants had a mean age of 20.2 (SD = 1.2); BMI of 21.6 (SD = 3.2); frequency of exercise/week of 3.0 (SD = 1.6); and duration of exercise/week of 63.2 minutes (SD = 37.7). The majority of the participants (98.7%) did not have any illnesses and were not on any medications.

### Measurement model of the DB-M

**Fig 1:**
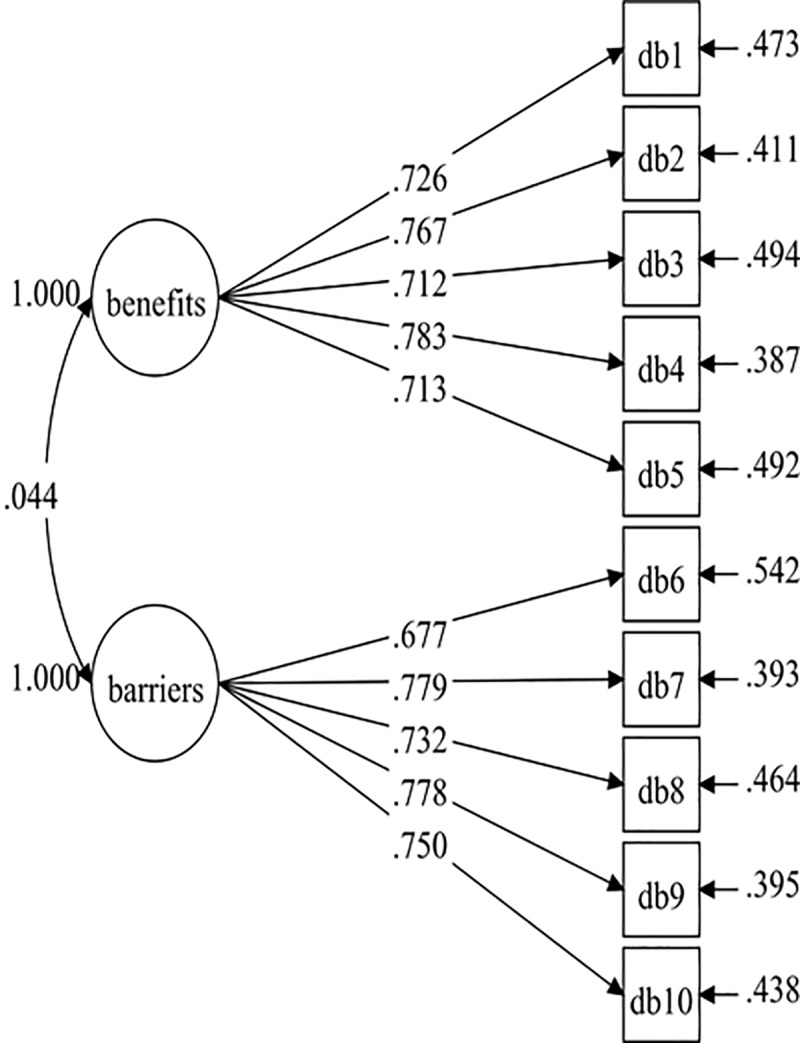
DB-M model (Model-1).

**Fig 2:**
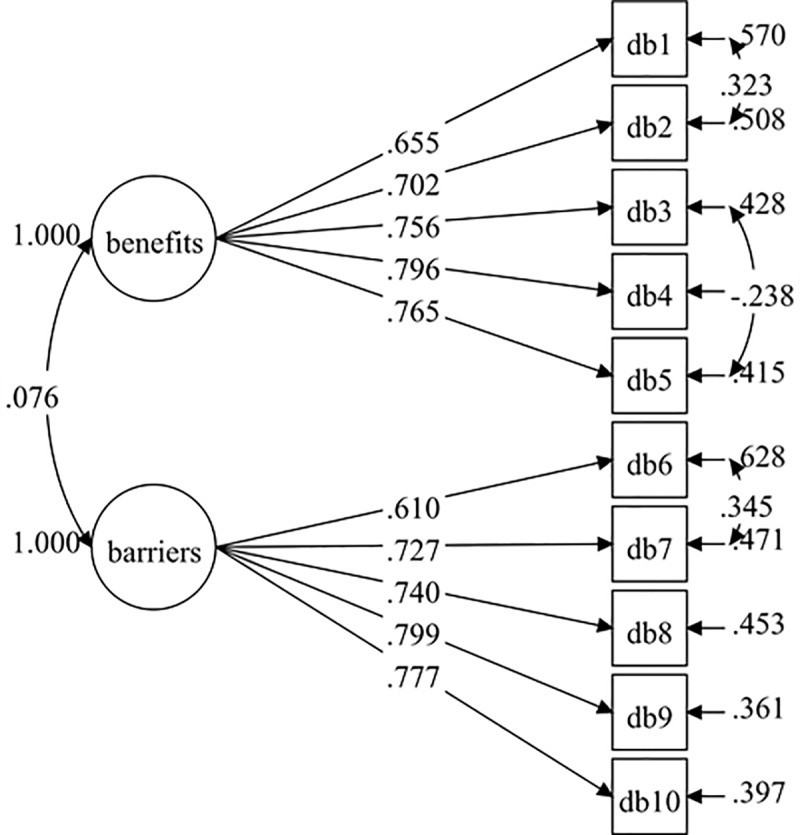
DB-M model (Model-2).

The hypothesised measurement model of the DB-M consisted of ten items and two factors (five items each). The initial model tested (Model 1) did not result in good fit indices (Table 1). However, all the standardised factor loadings were higher than .60 with p-value < .001 (Fig 1). The initial model (Model 1) was improved by adding three pairs of correlated residuals within the same factor based on the modification indices. The results (Model 2) showed a good fit with the data (Table 1). Therefore, Model 2 was established with adequate fit indices and standardised factor loadings of .610–.799 (Fig 2).

**Table 1:** Summary for DB-M model fit indices.

Path model	RMSEA (90% CI)	CFI	TLI	SRMR
Model-1	.075 (.064, .086)	.939	.919	.044
Model-2^a^	.047 (.034, .059)	.979	.969	.037

^a^Model-2 with correlated items residual; DB7 with DB6, DB2 with DB1, DB5 with DB3.

### Construct reliability and discriminant validity

The CR values of the final DB-M model (model 2) were .850 for perceived benefits and .839 for perceived barriers. The AVE values for perceived benefits and perceived barriers were .542 and .538, respectively. For discriminant validity, the correlation coefficient between the two factors (.027) was less than the recommended value of .85, indicating sufficient discriminant validity. Table 2 presents the CR, AVE values, and the factors correlation coefficient of the final DB-M model.

**Table 2:** Composite reliability [10], average variance extraction (AVE), factor correlation and squared correlation for DB-M final model.

Variables	CR (95% CI)	AVE	1	2
1. Benefits	.850 (.824, .876)	.542	1	.027
2. Barriers	.839 (.817, .862)	.538		1

Correlation p-value = .120.

### Measurement model of the DB-M for males and females

**Fig 3:**
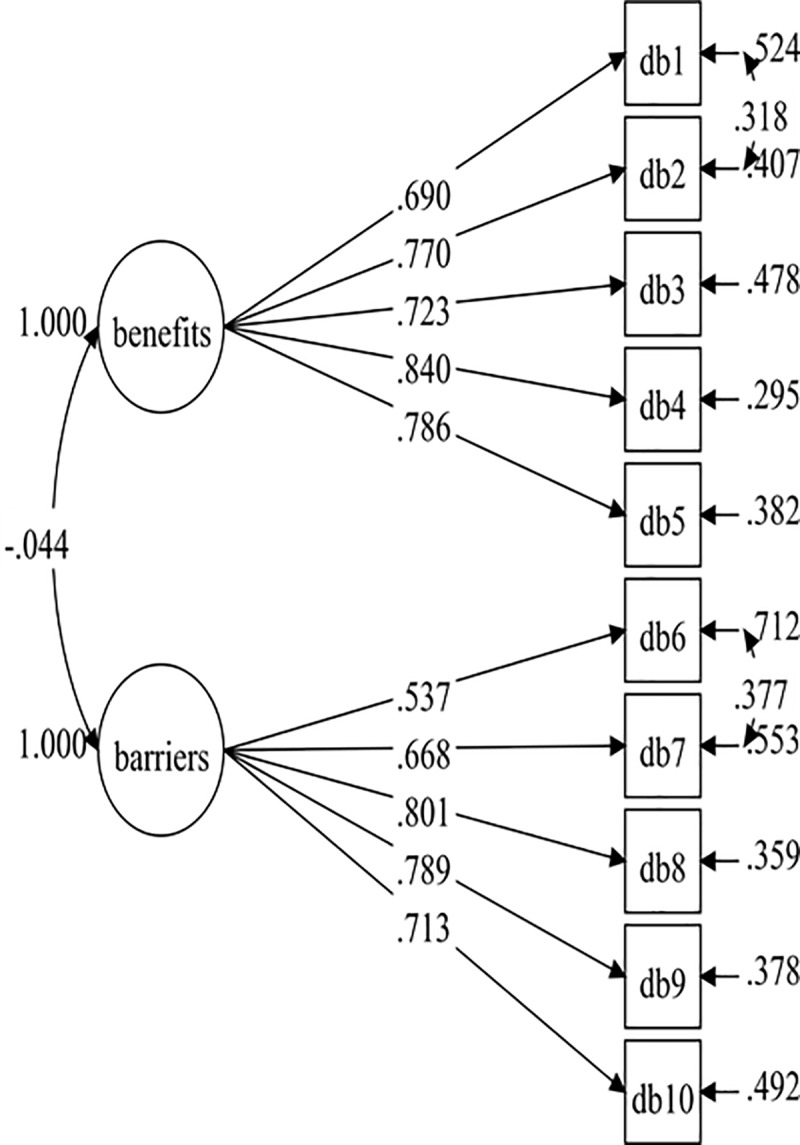
Male DB-M model (Model-4).

**Fig 4:**
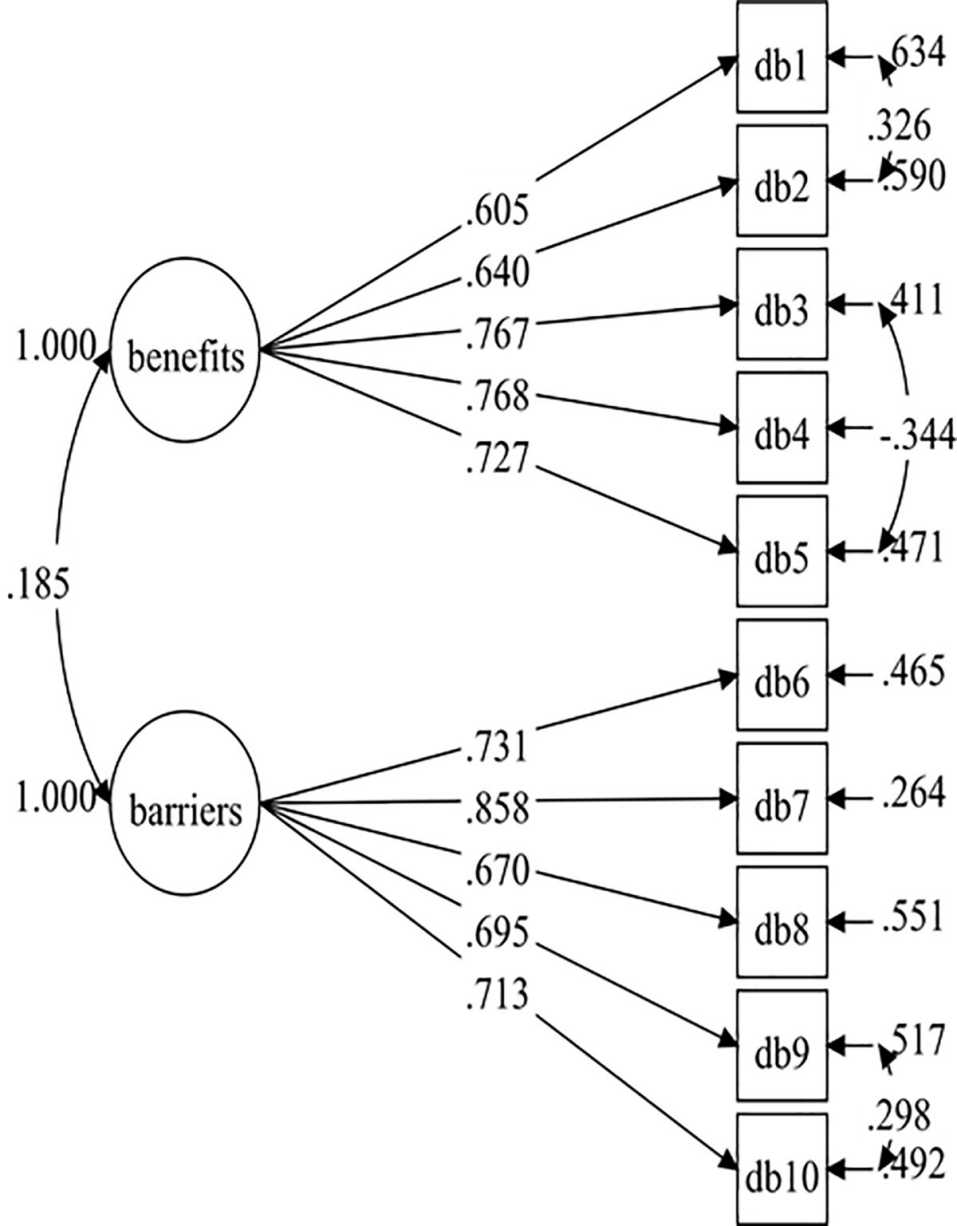
Female DB-M model (Model-6).

After establishing the overall baseline measurement model for all the data, the baseline measurement models for both males (Model 3) and females (Model 5) were specified. These two initial models did not result in a good fit with the data (Table 3). Therefore, both the male and female models have improved by adding correlated item residuals with the same factor for each model. The fit indices were adequate for both the final male model (Model 4: RMSEA = .052, CFI = .975, TLI = .964, SRMR = .040) and the final female model (RMSEA = .058, CFI = .965, TLI = .949, SRMR = .044). The final male model consisted of two added residual covariances (DB1 with DB2, and DB6 with DB7), and the final female model consisted of three added residual covariances (DB1 with DB2, DB9 with DB10, and DB3 with DB5). The baseline measurement models were not completely identical across groups (male and female). However, this differentiate in model specification should not affect model comparisons in other parameters in regard to invariance test [30, 31].The standardised factor loadings of the final male and female models were .537–.840 (Fig 3) and .605-.858 (Fig 4), respectively.

**Table 3:** The measurement model of DB-M baseline model fit results and tests of measurement and structure invariance.

Models	CFI	TLI	RMSEA	SRMR	Model comparison	ΔCFI	ΔTLI	ΔRMSEA
Model-3 (male group: hypothesised)	.933	.912	.081	.047	-	-	-	-
Model-4^b^ (male group: re-specified)	.975	.964	.052	.040	-	-	-	-
Model-5 (female group: hypothesised)	.927	.904	.079	.054	-	-	-	-
Model-6^c^ (female group: re-specified)	.965	.949	.058	.047	-	-	-	-
Model-7 (configural)	.970	.957	.055	.044	-	-	-	-
**Measurement invariance**								
Mode-8 (weak)	.965	.956	.055	.057	8 versus 7	-.005	-.001	0
Model-9 (strong)	.960	.955	.056	.062	9 versus 8	-.005	-.001	.001
Model-10 (strict)	.946	.945	.062	.065	10 versus 9	.014	-.010	.006
**Structural invariance**								
Model-11 (factor variance and covariance)	.957	.953	.057	.087	11 versus 9	-.003	-.002	.001
Model-12 (factor variance, covariance and factor mean)	.939	.934	.067	.104	12 versus 11	-.018	.-019	.010

^b^Adding residual covariances between item DB1 with DB2, DB7 with DB6.

^c^ Adding residual covariances between DB1 with DB2, DB10 with DB9, DB5 with DB3. Model-1 and Model-2 are reported in Table 1.

### Measurement and structural invariance

The fit indices for both the male and female sample model were within the recommended cut-off values by Hair et al. [29]. Hence, these two models were established with all the items retained. Firstly, the male and female models were integrated to establish the configural model invariance with the same number of fixed and free factor loadings. The configural invariance model fit the data well across gender (Table 3). Secondly, the weak measurement invariance model was established with adequate fit indices (Table 3). The weak measurement invariance model, when compared with the non-restrictive model (the configural model), resulted in adequate metric invariance differences across gender (ΔCFI = -.005, ΔTLI = -.001, ΔRMSEA = 0). This indicated that the items were interpreted similarly by both the male and female participants. Thirdly, the second most restrictive model (strong invariance model) was established. In the strong invariance model, equality restrictions were imposed on the factor loadings and item intercepts. These results (ΔCFI = -.005, ΔTLI = -.001, ΔRMSEA = .001) designated that the factor loadings and their intercepts remained invariant across gender. Finally, the highest most restrictive model (strict invariance model) was established. In the strict invariance model, equality restrictions were imposed on the factor loadings, item intercepts, and residual variances. These results (ΔCFI = .014, ΔTLI = .010, ΔRMSEA = .006) designated that the items’ average scores remained invariant across gender.

The structural invariance of the DB-M was assessed using the factor variance and factor covariance invariance, and the factor means invariance. The factor variance and factor covariance invariance fit the data well (CFI = .957, TLI = .953, SRMR = .087, RMSEA = .057), and its differences with the less restrictive invariance model (strong invariance) are within the acceptable values (ΔCFI = -.003, ΔTLI = -.002, ΔRMSEA = .001). These results signified that the relationships among the two factors of the DB-M remained the same across gender. The factor means invariance fit indices were slightly below the recommended values (CFI = .939, TLI = .934, SRMR = .104, RMSEA = .067), and its differences with the less-restrictive model (factor variance and covariance) are slightly below the recommended values (ΔCFI = -.018, ΔTLI = -.019, ΔRMSEA = .010). This result signals that the factor means are not invariant across gender.

### Internal consistency

The Cronbach’s alpha values were .857 for perceived benefits, and .859 for perceived barriers. The item-total correlation was .613–.716. According to Nunnally and Bernstein [38], the item-total correlation of greater than .30 signifies sufficient internal consistency, and each item contributes adequately to the measurement of its factor.

### Test-retest reliability

For test-retest reliability, 100 participants (female: 83, male: 17) volunteered to complete the DB-M again on day 14. The mean score for perceived benefits decreased from 21.0 (SD = 3.1) to 19.8 (SD = 3.3), with an ICC value of .979 (95% CI, .969, .986, p-value < .001). The mean score for perceived barriers increased from 10.4 (SD = 3.2) to 11.6 (SD = 3.9), with an ICC value of .960 (95% CI, .941, .973, p-value < .001). Based on gender difference: the ICC for perceived benefits was .975 (95% CI: .933, .991, p-value < .001) for males and .980 (95% CI: .969, .987, p-value < .001) for females, and the ICC for perceived barriers was .944 (95% CI: .854, .980, p-value < .001) for males and .962 (95% CI: .941, .975, p-value < .001) for females. These ICC values revealed that the DB-M had an excellent stability over time [39].

## Discussion

The present study aimed to translate the English version of the DB scale into Malay (DB-M) and then evaluate its validity with university students using CFA. This study presented evidence for adequate psychometric properties of the DB-M scale consistent with previous studies [14, 37]. The DB-M scale fit the data well, and the final model retained all ten items with strong factor loadings (above .60) on their respective factors. Furthermore, the results provided substantial evidence for measurement and structural invariance across gender.

The DB-M demonstrated adequate construct validity and discriminant validity. The CR and variance extracted of the DB-M, or the degree to which the items revealed their respective factors, exceeded the prescribed values of .60 for CR [31] and .50 for AVE [32]. These results showed that the DB-M had adequate construct validity, and all the items accurately estimate their respective factors. For discriminant validity of the DB scale, the correlation coefficient between the two factors (.027) was less than the prescribed value of .85 [33]. These results showed sufficient discriminant validity of the DB scale, with each factor explaining distinct information from the other factor.

According to the TTM, individuals need many struggles at the behavioural change to be successful [40]. The decisional balance is among the psychological constructs of the TTM that influences exercise behaviour [41]. Therefore, it is necessary to ascertain an instrument that reliably assesses the decisional balance constructs. The DB-M model tested in the present study reveals sufficient internal consistency. The Cronbach’s alpha values of .857 for perceived benefits and .859 for perceived barriers matches the previous studies Cronbach’s of .79 and .71 [14], and .87 and .70 [37], for perceived benefits and perceived barriers, respectively. Additionally, the ICC values for test-retest reliability (.979 for perceived benefits and .960 for perceived barriers) computed in this study indicate that the DB-M scale has excellent stability over time [39]. A previous study reported a 2-weeks test-retest reliability coefficient of .91 and .89 for perceived benefits and perceived barriers, respectively [41].

The present study investigated the measurement invariance of the DB-M across gender and ascertained that all the invariance requirements were met [26, 42]. These results signified that the male and female samples had similar understandings of all the ten items in the DB-M, which is vital when making valid comparisons between the male and female students’ decisional balance to exercise. Furthermore, the structural invariance of the DB-M was desirable for factor variance and covariance, but the factor means were not desirable (variant) across gender. These results indicate that the relationships between the factors remain the same across gender, but the means of the factors varies across the gender. A previous study by McAuley et al. [43] reported that males have higher confidence than females in completing exercise prescription.

Residual covariances may be specified as a means to examine hypotheses regarding shared sources of variability over and beyond the factors [31]. In the present study, covariances between residuals’ items within the same factor were added to improve the model fit indices. These modifications on the hypothesized DB-M model were determined based on the MI values reported in Mplus output and after sufficient theoretical support was established by the researchers. Covariance between residuals for items DB1 (Physical activity would help me reduce tension or manage stress) and DB2 (I would feel more confident about my health by getting physical activity) were added to both the final overall model, male model, and female model. This is reasonable, as previous studies reported a strong relationship between mental health, stress, mood and physical exercise [44] Other covariances added were the error residual for DB6 (I am too tired to get physical activity because of my other daily responsibilities) and DB7 (Physical activity would take too much of my time) for the final overall model and male model, the error residual for DB3 (I would sleep better) and DB5 (Physical activity would help me control my weight) for the final overall model and female model, and the error residual for DB9 (I’d worry about looking awkward if others saw me being physically active) and DB10 (Getting physical activity would cost too much money) for the final female model only. A better fitting model was established after adding these residual covariances. In social psychological research, when these covariances make a substantive sense, they should be included in the model [45, 46].

In this study, some study limitations exist, and recommendations for future research are needed. First, although the sample size for this study is considered to be large, caution must be given to the generalisability of the results, as the data were collected from a single university. Second, the self-reported measure was used to assess the student’s decisional balance to exercise. Self-reported measures have been associated with response bias, which could reduce the accuracy of the data obtained. Nevertheless, the questions were answered anonymously, and the participants were assured of their confidentiality. Also, future research should examine the replicability of the DB-M with a more diverse Malaysians population of different ages, education levels, occupations, and health conditions.

## Conclusion

In the present study, the DB-M was shown to have adequate psychometric properties and can be administered to evaluate decisional balance for exercise behaviour among university students. All the items were retained with strong factor loadings. Also, the scale displayed sufficient measurement invariance (configural, weak, strong, and strict) and structural invariance (factor variance and covariance). These findings illustrate that DB-M can be used to make valid comparisons across gender.

## Supporting information

S1 Data (PDF)

S2 Data (SAV)
