# Supplementary material for: Factorial validity, measurement and structure invariance of the Malay language decisional balance scale in exercise across gender
Source: PLoS One. 2020 Mar 18;15(3):e0230644. doi: 10.1371/journal.pone.0230644 (PMC7080256; doi:10.1371/journal.pone.0230644)
Supplement: S1 Data — (PDF) [file pone.0230644.s001.pdf]

ID	Gender	Age	Ethnic	Sport_frequency	Sport_duration	DB1
1	1	20	2	2	120	3
2	2	20	1	3	60	5
3	2	20	2	2	60	3
4	2	22	1	3	60	4
5	1	20	2	2	60	2
6	2	21	3	2	120	5
7	2	22	1	3	120	3
8	2	20	2	2	120	3
9	2	20	3	3	60	4
10	1	21	2	4	120	3
11	2	23	1	3	60	2
12	2	21	2	6	90	3
13	1	19	1	7	120	4
14	1	19	1	4	40	5
15	2	19	1	1	30	3
16	1	19	3	3	23	5
17	2	21	1	3	100	3
18	1	20	2	7	60	4
19	1	21	2	3	90	4
20	2	20	2	3	60	5
21	1	21	2	1	120	4
22	2	21	2	2	30	4
23	2	21	2	7	60	3
24	2	21	2	3	30	4
25	2	19	4	2	180	5
26	1	21	2	3	60	4
27	1	21	2	1	30	5
28	2	20	2	2	120	3
29	2	20	2	7	30	4
30	1	21	2	3	60	3
31	2	19	2	2	120	5
32	1	20	2	2	120	3
33	2	20	3	1	120	3
34	2	22	2	7	90	3
35	2	22	3	3	120	4
36	2	20	2	4	30	3
37	2	20	2	1	60	4
38	1	19	2	2	60	4
39	2	21	2	2	15	5
40	2	20	2	2	30	3
41	2	19	2	4	30	5
42	2	20	2	2	90	4
43	2	20	2	1	120	3
44	2	19	4	2	90	3
45	2	19	3	2	60	4
46	2	20	2	2	90	4
47	2	20	2	2	60	3
48	1	20	2	12	30	5
49	2	19	2	2	60	4
50	1	20	3	2	60	4
51	2	20	3	3	15	5
52	1	20	1	1	60	3
53	1	20	1	4	260	4
54	1	20	1	3	20	2
55	1	21	1	4	60	4

56	1	20	1	1	120	4
57	2	19	3	2	30	5
58	2	20	3	1	120	2
59	2	21	2	2	15	5
60	1	21	3	3	120	3
61	2	20	2	7	120	5
62	1	22	3	3	120	4
63	2	20	3	2	30	3
64	2	21	3	5	60	4
65	2	21	2	1	30	3
66	1	19	1	4	60	2
67	2	21	2	7	30	4
68	2	20	2	1	30	4
69	2	21	3	2	15	3
70	2	22	3	3	15	3
71	2	23	2	3	30	3
72	1	22	2	3	45	4
73	2	21	2	2	60	4
74	2	20	2	2	45	4
75	2	19	2	3	60	5
76	1	20	2	2	45	4
77	1	19	2	2	60	5
78	2	19	2	2	60	2
79	1	21	1	3	45	3
80	1	20	1	1	50	3
81	2	20	2	2	60	5
82	2	21	2	6	20	3
83	1	21	2	3	30	3
84	2	20	2	7	13	4
85	2	21	4	4	120	5
86	2	21	2	2	30	3
87	2	22	2	1	20	4
88	2	20	4	3	60	4
89	1	20	1	5	60	2
90	1	22	1	2	20	4
91	1	21	1	7	60	4
92	2	20	1	3	15	3
93	2	20	1	1	120	5
94	1	20	1	4	90	4
95	1	20	1	5	30	5
96	2	20	2	3	60	4
97	2	20	2	2	50	3
98	2	19	2	3	30	4
99	2	20	2	2	30	4
100	2	20	2	1	20	3
101	1	20	2	14	10	3
102	2	20	2	3	30	2
103	1	20	2	2	180	2
104	1	20	2	2	30	4
105	1	20	2	1	15	3
106	2	20	2	1	60	5
107	2	20	2	2	30	3
108	1	20	2	4	45	5
109	2	19	3	2	20	4
110	2	20	2	3	30	4
111	2	20	3	4	60	4

112	2	20	2	7	60	3
113	2	19	3	2	60	4
114	2	22	2	2	30	4
115	2	20	3	7	120	3
116	2	20	2	7	45	5
117	2	20	2	2	100	4
118	2	19	3	2	40	4
119	2	20	1	2	120	4
120	1	21	1	5	120	5
121	2	20	3	2	30	4
122	1	21	1	3	60	4
123	2	20	3	3	25	4
124	2	24	1	2	30	4
125	2	20	2	4	120	3
126	2	20	2	1	120	3
127	1	22	1	2	30	4
128	2	21	3	2	90	4
129	1	21	3	5	90	4
130	1	23	2	3	15	4
131	2	21	2	3	120	5
132	2	20	1	2	90	5
133	2	22	2	3	60	5
134	2	22	1	5	60	4
135	2	19	1	4	60	4
136	2	19	1	7	20	4
137	2	19	1	2	30	4
138	2	19	1	2	60	3
139	1	21	1	1	60	3
140	1	22	1	5	60	4
141	1	19	1	2	60	2
142	2	19	1	2	60	4
143	1	19	1	2	120	4
144	1	21	1	4	120	3
145	2	18	1	1	30	3
146	2	18	1	3	30	4
147	2	19	3	2	120	5
148	2	20	2	2	60	5
149	2	19	3	2	90	3
150	2	20	1	3	30	5
151	2	20	3	3	45	5
152	1	22	1	2	60	3
153	2	18	1	3	45	4
154	2	22	2	3	60	5
155	1	20	1	4	60	5
156	2	20	3	4	30	4
157	1	21	1	5	60	3
158	1	20	1	7	120	5
159	2	19	1	2	30	4
160	2	20	1	2	30	3
161	2	21	1	4	60	4
162	2	19	1	6	90	4
163	2	20	1	3	45	4
164	2	20	3	2	120	4
165	2	20	3	2	60	4
166	2	19	1	4	120	4
167	2	19	1	4	120	4

168	1	20	1	2	120	3
169	1	18	1	3	45	3
170	1	21	1	5	70	4
171	1	21	4	4	60	4
172	1	19	1	3	60	2
173	2	20	4	2	30	4
174	2	21	2	3	40	4
175	1	20	1	2	30	3
176	1	22	1	5	90	4
177	1	22	1	2	120	4
178	1	22	1	2	90	4
179	1	22	1	4	90	4
180	2	20	1	1	75	4
181	1	18	1	4	60	5
182	2	20	3	2	75	2
183	2	20	1	3	30	4
184	2	20	1	6	120	4
185	2	20	1	2	20	5
186	2	20	1	3	50	4
187	2	20	4	2	10	4
188	2	20	3	4	60	5
189	2	19	4	2	105	5
190	2	19	2	1	30	3
191	1	19	1	3	40	5
192	1	20	2	3	120	3
193	2	19	3	1	60	3
194	1	19	1	4	75	5
195	1	19	1	2	30	4
196	1	19	1	5	35	5
197	1	19	1	1	45	5
198	1	19	1	5	45	3
199	1	19	1	7	90	5
200	1	19	1	5	45	3
201	1	19	1	7	35	4
202	1	20	2	1	120	3
203	1	20	1	1	10	4
204	2	24	3	2	120	4
205	2	19	4	3	60	5
206	1	20	3	3	60	5
207	2	19	1	3	60	5
208	1	19	1	4	120	3
209	1	21	2	2	60	2
210	1	22	1	4	90	5
211	1	22	1	3	90	4
212	2	21	3	2	60	5
213	2	21	3	2	90	3
214	1	22	1	4	60	5
215	1	22	1	7	60	4
216	2	20	2	2	30	4
217	2	19	2	1	30	4
218	2	19	4	2	90	4
219	2	19	2	3	90	2
220	1	19	2	1	30	4
221	2	19	2	3	10	4
222	2	19	2	2	120	5
223	1	19	2	2	120	5

224	2	20	2	2	30	4
225	1	21	1	2	30	5
226	1	19	1	3	60	4
227	1	21	1	3	15	5
228	2	19	1	3	60	5
229	1	21	1	1	30	1
230	1	20	1	4	90	4
231	1	23	4	3	90	5
232	1	20	2	3	10	5
233	2	19	1	3	30	4
234	2	19	1	2	60	4
235	2	20	1	2	90	4
236	1	20	1	5	100	5
237	1	20	1	3	20	1
238	2	20	2	3	30	4
239	1	21	1	4	60	4
240	2	20	1	3	15	3
241	2	20	3	2	60	3
242	1	21	1	7	60	4
243	1	20	1	5	30	5
244	1	20	1	4	90	4
245	2	20	3	1	60	4
246	2	20	3	7	180	4
247	2	21	3	5	60	5
248	2	21	3	3	45	5
249	1	21	1	2	90	5
250	2	19	1	1	10	4
251	1	22	1	7	120	5
252	1	20	1	3	120	5
253	2	19	1	3	120	4
254	2	23	1	2	60	5
255	2	18	1	2	60	4
256	1	19	1	2	60	5
257	1	19	1	4	60	4
258	1	19	1	6	70	4
259	2	19	1	2	60	1
260	2	19	4	5	45	4
261	1	22	3	3	90	4
262	2	23	1	2	60	5
263	1	23	1	4	60	4
264	1	22	1	3	45	5
265	2	19	1	2	60	4
266	1	20	1	2	45	4
267	2	20	1	2	20	5
268	2	20	1	2	60	5
269	2	20	1	1	45	2
270	2	20	1	2	30	3
271	2	20	1	3	30	4
272	2	19	1	2	15	4
273	2	21	1	3	30	3
274	2	19	1	1	30	3
275	2	19	1	3	30	5
276	2	20	1	1	45	3
277	2	20	1	2	30	4
278	1	21	1	3	60	5
279	1	21	1	2	30	3

280	1	21	1	2	60	5
281	2	19	2	2	30	4
282	2	21	2	2	20	4
283	2	21	1	3	30	3
284	2	20	4	2	10	4
285	1	21	1	4	60	5
286	2	20	1	3	30	4
287	1	21	1	7	90	4
288	1	19	1	6	90	4
289	1	21	1	2	30	4
290	1	22	1	4	15	3
291	1	20	1	2	60	2
292	1	19	2	4	50	3
293	1	21	1	3	60	4
294	2	21	1	2	60	3
295	1	21	1	2	60	2
296	2	20	1	1	30	5
297	2	21	1	3	20	3
298	2	21	1	4	15	5
299	2	21	1	2	21	5
300	2	21	1	4	30	4
301	1	20	2	3	40	4
302	2	21	1	2	30	4
303	2	21	1	2	60	4
304	2	21	1	2	60	4
305	2	21	1	3	120	5
306	1	21	2	2	60	2
307	2	20	4	2	30	4
308	2	20	1	3	180	4
309	2	20	1	2	30	3
310	2	20	2	3	60	3
311	1	20	1	7	60	4
312	2	21	3	3	30	4
313	2	22	1	3	30	5
314	2	19	3	2	120	5
315	2	19	1	1	180	5
316	2	18	1	1	30	3
317	1	20	1	4	60	5
318	2	20	3	4	30	4
319	2	19	1	2	60	4
320	2	19	1	4	15	3
321	1	19	1	5	60	3
322	1	19	1	2	60	4
323	2	19	1	2	90	3
324	2	19	1	3	30	4
325	1	21	1	1	60	5
326	2	20	1	4	30	4
327	2	20	4	3	30	4
328	2	19	1	2	60	1
329	1	21	1	5	120	5
330	1	20	1	2	90	5
331	2	21	3	3	20	4
332	2	22	4	2	40	3
333	1	20	1	5	90	4
334	2	20	3	5	45	4
335	1	21	1	1	150	4

336	1	20	1	5	45	3
337	1	23	1	4	90	5
338	2	21	4	1	30	5
339	2	21	1	1	30	4
340	2	19	4	1	60	4
341	2	19	1	3	90	4
342	1	23	2	3	90	4
343	2	19	1	5	60	4
344	2	19	1	3	30	4
345	2	20	1	3	75	4
346	2	19	1	3	45	4
347	2	20	1	3	60	4
348	1	24	4	3	50	5
349	1	19	1	3	75	5
350	1	19	1	4	60	5
351	1	22	1	3	60	3
352	1	19	1	1	120	2
353	1	19	1	2	20	3
354	1	20	1	4	60	4
355	2	19	1	4	60	4
356	2	21	1	3	30	4
357	2	19	1	3	25	4
358	2	19	1	2	30	4
359	1	21	1	2	120	5
360	1	23	1	4	60	3
361	1	19	1	2	90	4
362	1	19	1	1	15	3
363	2	19	1	2	60	2
364	1	18	1	1	180	4
365	2	21	2	2	80	5
366	2	19	1	3	100	4
367	1	19	1	3	180	4
368	1	20	1	3	180	4
369	2	24	3	4	50	3
370	2	21	1	2	30	4
371	2	21	1	1	30	4
372	2	20	1	2	90	3
373	2	19	4	1	60	4
374	2	20	1	4	120	4
375	2	21	4	2	45	3
376	2	20	4	2	30	3
377	1	22	3	2	30	4
378	2	20	4	1	90	4
379	1	20	1	6	37	4
380	2	21	1	3	45	4
381	2	20	4	3	90	3
382	2	20	4	3	60	3
383	2	20	4	2	20	3
384	2	19	4	3	30	4
385	2	19	4	3	60	5
386	2	18	4	2	30	3
387	2	19	4	2	45	4
388	1	19	1	3	120	4
389	2	19	2	1	60	4
390	2	19	2	2	90	3
391	1	20	1	3	120	3

392	2	20	2	2	60	4
393	2	19	1	1	10	3
394	1	20	1	1	60	3
395	2	19	1	2	90	3
396	2	20	4	2	30	3
397	1	21	2	2	60	2
398	1	20	1	1	15	4
399	2	19	1	2	60	5
400	2	20	3	3	30	2
401	2	19	3	2	45	4
402	1	21	1	2	30	3
403	2	19	1	2	180	4
404	1	19	1	6	45	4
405	1	19	1	7	60	4
406	2	19	1	3	30	4
407	2	18	1	2	30	4
408	1	22	1	2	90	4
409	1	19	1	2	60	4
410	2	19	1	2	30	4
411	2	19	1	5	120	4
412	2	19	1	2	60	4
413	2	19	1	5	60	4
414	2	20	1	1	30	5
415	2	19	1	3	90	4
416	2	19	1	3	60	3
417	2	20	4	2	90	4
418	1	20	1	3	30	4
419	1	20	1	7	120	4
420	2	19	1	3	30	3
421	2	20	1	1	120	3
422	1	20	1	2	45	3
423	2	19	1	3	90	4
424	2	20	1	5	60	4
425	2	20	1	2	30	4
426	1	20	1	5	45	5
427	2	19	1	2	30	4
428	2	20	1	2	30	3
429	2	20	1	1	20	4
430	2	20	1	1	60	5
431	2	22	1	2	30	4
432	2	20	1	3	30	3
433	2	20	1	2	10	3
434	1	20	1	4	30	3
435	2	20	1	2	60	3
436	2	20	1	3	120	3
437	2	19	1	1	15	3
438	2	19	1	3	60	5
439	1	19	4	3	50	3
440	2	19	4	2	20	4
441	1	19	1	2	80	4
442	1	21	1	3	60	4
443	1	21	1	3	120	5
444	1	21	1	3	120	5
445	1	21	1	3	120	4
446	1	21	1	3	120	3
447	1	21	1	3	120	5

448	1	20	1	2	60	4
449	2	22	1	1	20	4
450	1	20	1	2	120	4
451	2	20	2	2	60	4
452	2	20	3	2	60	5
453	2	20	2	2	60	4
454	2	20	3	1	60	3
455	1	20	1	2	60	4
456	1	20	1	1	120	4
457	2	20	1	3	90	2
458	1	22	1	5	30	5
459	1	19	1	3	1	4
460	1	21	1	1	30	4
461	1	19	1	3	60	4
462	1	19	1	3	60	4
463	2	23	2	2	60	5
464	2	20	1	2	90	4
465	2	22	2	3	60	4
466	1	19	1	3	60	4
467	1	20	1	1	10	5
468	1	19	1	2	60	3
469	1	20	3	3	60	5
470	2	20	1	3	30	4
471	1	19	1	2	60	3
472	2	20	1	2	45	4
473	1	20	1	4	50	4
474	1	20	1	3	60	2
475	2	20	1	2	45	3
476	1	20	1	2	90	5
477	2	20	1	1	60	4
478	2	20	1	1	45	3
479	1	20	1	1	45	4
480	1	20	1	2	60	3
481	1	22	1	1	60	4
482	2	21	1	3	10	3
483	1	21	1	2	30	3
484	1	23	4	4	45	4
485	2	19	4	4	90	4
486	1	19	1	2	60	5
487	2	20	2	3	60	5
488	2	19	1	2	120	5
489	2	19	1	2	120	4
490	2	19	1	1	30	3
491	2	21	1	3	30	3
492	2	18	2	2	60	2
493	2	18	2	1	20	5
494	1	22	2	3	75	4
495	1	19	2	4	90	4
496	2	19	2	4	90	4
497	2	19	2	1	20	4
498	2	19	2	2	45	4
499	2	19	2	2	60	3
500	2	22	2	2	30	4
501	2	20	1	2	45	4
502	2	20	1	5	90	4
503	2	19	1	1	60	5

504	1	19	1	2	60	4
505	1	19	1	5	60	4
506	1	20	1	2	30	4
507	1	19	1	4	60	3
508	1	19	1	3	75	5
509	1	19	1	2	40	4
510	1	19	1	4	75	4
511	1	19	1	3	60	4
512	2	21	1	2	30	5
513	2	19	1	2	90	4
514	2	21	2	3	45	2
515	2	22	2	4	45	2
516	1	20	1	3	75	2
517	1	20	1	6	45	3
518	1	20	2	4	75	3
519	2	22	1	4	10	3
520	2	19	1	4	60	5
521	1	20	1	3	60	5
522	1	22	1	3	60	5
523	1	19	1	3	30	4
524	2	20	1	2	60	5
525	1	19	1	2	60	3
526	1	19	2	4	120	4
527	1	19	1	3	60	4
528	1	19	1	1	60	3
529	2	22	1	3	60	4
530	2	20	1	3	60	5
531	2	19	1	3	60	3
532	2	20	3	2	30	4
533	1	19	3	5	60	4
534	2	24	1	3	30	5
535	2	20	1	1	30	5
536	2	19	3	2	30	4
537	1	18	1	2	60	3
538	1	19	1	2	30	3
539	1	20	1	3	60	2
540	1	19	1	3	90	4
541	1	19	1	3	90	3
542	2	19	1	4	60	5
543	1	19	1	3	90	4
544	1	19	1	4	90	4
545	1	19	1	3	60	5
546	2	19	1	2	60	4
547	1	19	1	3	90	4
548	1	20	1	4	90	4
549	1	19	1	3	90	4
550	1	19	3	4	60	3
551	2	24	4	1	30	2
552	2	20	1	1	20	4
553	2	21	2	1	30	4
554	2	19	1	1	30	4
555	1	20	1	4	60	4
556	1	20	1	5	60	4
557	1	21	1	2	60	4
558	1	21	1	4	60	5
559	1	21	1	2	60	4

560	2	20	1	1	30	4
561	2	20	1	2	30	4
562	2	20	1	2	30	4
563	2	20	1	1	15	5
564	2	21	2	1	30	4
565	2	20	3	2	30	5
566	1	22	1	3	90	5
567	2	19	1	2	60	3
568	2	19	3	2	90	4
569	2	19	3	3	25	5
570	1	20	2	2	60	5
571	1	20	4	1	30	4
572	1	21	1	2	120	5
573	2	21	2	1	60	4
574	2	21	2	7	30	4
575	2	21	2	7	30	3
576	2	21	2	1	60	3
577	1	22	1	2	90	4
578	1	20	1	4	120	4
579	1	19	1	5	120	3
580	2	19	1	1	30	3
581	2	22	1	2	30	5
582	2	22	1	3	180	4
583	2	19	2	2	90	4
584	1	20	1	1	40	3
585	2	23	1	2	15	4
586	2	22	2	3	30	4
587	2	19	1	4	20	5
588	1	22	2	2	20	3
589	2	19	1	3	30	5
590	2	22	3	2	15	3
591	2	24	1	1	60	3
592	1	23	1	3	30	4
593	1	20	1	7	120	5
594	1	20	1	7	120	4
595	1	20	1	7	120	4
596	1	21	1	2	120	3
597	2	23	1	1	90	5
598	1	21	1	3	60	4
599	2	19	1	2	45	4
600	1	20	1	2	40	4
601	2	21	2	2	60	4
602	1	20	1	1	60	4
603	1	20	1	4	120	4
604	1	21	1	5	160	4
605	1	22	1	5	120	4
606	1	20	1	2	120	3
607	1	20	1	1	30	2
608	2	20	1	1	40	4
609	1	21	1	2	60	2
610	1	23	1	2	30	4
611	1	22	1	3	60	4
612	2	23	1	3	30	4
613	1	21	2	2	60	2
614	1	24	4	7	30	3
615	1	20	1	3	120	4

616	2	21	1	2	60	4
617	1	20	1	2	120	4
618	1	20	1	2	30	4
619	1	21	1	2	120	4
620	1	20	1	5	60	4
621	2	22	1	3	120	4
622	2	20	1	1	30	4
623	2	22	1	3	120	4
624	1	21	2	2	60	2
625	1	21	1	1	120	2
626	1	23	3	1	40	3
627	1	21	1	2	60	4
628	2	22	1	2	40	4
629	1	22	1	6	90	4
630	1	23	2	1	28	5
631	1	20	1	6	40	4
632	1	20	2	5	60	4
633	2	19	1	4	90	4
634	2	22	1	3	120	3
635	1	19	1	5	90	4
636	1	20	1	6	60	4
637	1	19	1	5	60	5
638	1	19	1	5	60	3
639	1	20	1	4	60	2
640	1	21	1	3	45	2
641	2	19	1	5	60	4
642	1	20	1	4	60	4
643	1	19	1	3	90	3
644	1	19	1	3	60	4
645	1	19	1	2	160	4
646	1	19	1	7	30	3
647	1	19	1	3	120	4
648	1	22	1	7	15	4
649	1	22	1	2	60	3
650	1	19	1	4	180	3
651	1	19	1	3	90	2
652	1	19	1	3	60	2
653	1	19	1	4	60	5
654	1	19	1	6	90	5
655	1	19	1	1	150	4
656	1	21	4	5	70	5
657	1	21	1	6	120	5
658	1	24	4	4	30	5
659	1	21	1	5	90	4
660	1	21	1	3	60	5
661	1	21	1	5	45	4
662	1	19	1	2	30	3
663	1	20	4	5	30	5
664	1	19	4	2	60	4
665	2	19	1	2	45	4
666	2	19	1	3	60	4
667	1	20	2	3	180	3
668	2	23	1	2	20	3
669	2	21	1	1	60	3
670	1	23	1	5	60	4
671	1	22	2	2	20	3

672	2	20	2	2	60	3
673	2	20	2	4	240	3
674	1	22	1	2	90	4
675	1	20	1	3	35	4
676	1	22	2	2	60	5
677	1	23	2	4	40	4
678	1	20	1	7	120	5
679	1	21	1	5	60	4
680	1	21	1	3	60	4
681	2	20	3	3	30	5
682	2	19	2	1	10	5
683	1	20	1	4	60	4
684	1	20	1	7	120	3
685	1	21	1	6	60	3
686	1	20	1	4	30	5
687	1	21	1	3	45	3
688	1	20	1	5	10	5
689	1	21	1	3	30	3
690	1	20	1	5	30	3
691	1	22	1	5	45	3
692	1	20	3	5	30	3
693	1	20	1	5	40	3
694	1	20	1	2	45	3
695	1	20	1	2	60	2
696	1	21	1	3	30	2
697	1	20	1	7	120	4
698	1	24	3	7	180	1
699	1	20	1	3	90	3
700	1	19	1	4	50	5
701	1	19	1	4	60	5
702	1	19	1	5	25	4
703	1	19	1	3	60	4
704	1	19	1	3	60	4
705	1	19	1	4	30	4
706	1	19	1	4	60	4
707	1	19	1	3	60	4
708	1	19	1	6	90	4
709	1	19	1	5	60	5
710	1	19	1	5	60	4
711	2	21	1	3	30	4
712	2	23	3	2	25	4
713	2	20	1	2	120	4
714	1	22	1	4	120	5
715	2	20	1	1	30	5
716	2	21	2	3	60	4
717	2	23	2	1	120	4
718	2	21	1	1	180	4
719	2	19	1	2	30	5
720	1	19	1	1	180	4
721	2	19	1	2	30	5
722	1	19	1	2	70	5
723	1	22	1	1	180	4
724	2	20	1	3	30	5
725	1	24	2	5	120	3
726	1	20	2	3	30	4
727	1	24	2	4	30	4

728	2	22	2	5	30	4
729	2	19	2	2	90	4
730	2	22	2	3	30	4
731	1	21	2	2	120	4
732	1	19	1	4	90	4
733	1	19	1	5	60	3
734	1	19	1	4	40	4
735	1	19	1	3	120	4
736	1	24	3	3	60	4
737	1	20	1	3	120	3
738	1	22	1	3	120	5
739	2	21	2	2	60	2
740	2	19	3	2	60	4
741	2	19	2	2	60	3
742	1	19	1	1	60	3
743	2	20	2	1	60	2
744	2	19	2	3	90	3
745	2	20	4	1	30	5
746	1	19	4	5	30	5
747	1	19	1	3	120	4
748	1	20	1	5	120	5
749	2	20	3	3	20	4
750	1	22	3	2	60	4

DB2	DB3	DB4	DB5	DB6	DB7	DB8
4	3	4	2	4	4	3
3	3	3	3	5	4	4
4	2	2	4	3	2	4
4	3	3	2	4	2	3
5	4	2	3	5	5	4
2	4	4	3	3	2	3
3	5	5	3	3	3	2
3	4	4	5	3	5	4
4	5	5	3	4	2	3
2	3	3	3	3	3	3
3	4	4	3	2	5	2
3	3	5	4	4	3	4
4	4	4	4	2	3	3
5	5	5	5	1	2	2
4	3	4	3	4	4	4
5	5	5	4	3	3	4
4	5	4	4	3	3	5
2	4	2	2	4	3	3
5	3	5	4	2	3	2
5	3	3	4	4	4	1
4	3	4	3	4	3	2
4	4	3	4	3	3	3
2	3	2	3	3	3	3
3	3	3	1	3	1	3
4	3	3	2	5	3	2
4	5	5	5	3	2	2
4	3	1	2	4	2	1
4	3	4	5	4	5	5
4	5	4	3	3	2	3
4	4	3	3	4	5	2
4	3	4	3	2	1	2
4	3	4	4	4	3	3
3	2	4	3	3	2	1
4	4	5	5	5	4	2
4	5	5	5	4	3	3
4	2	3	3	2	3	3
4	4	4	4	3	3	3
4	3	5	4	2	1	2
5	5	5	4	5	4	5
4	3	3	5	5	5	3
1	4	5	2	4	3	3
4	3	3	4	4	4	3
4	3	4	4	2	3	4
4	4	3	3	3	3	3
4	4	4	3	2	1	1
4	5	4	3	3	2	4
4	3	4	4	3	3	3
4	4	4	4	3	3	1
4	4	4	3	3	2	2
4	5	5	5	3	4	4
5	4	5	5	1	1	1
3	4	4	4	3	3	4
4	4	4	4	4	4	4
3	4	4	4	5	3	1
4	4	4	4	3	3	2

4	4	4	4	4	4	4
5	4	5	5	2	1	5
3	3	4	4	3	2	1
4	5	5	5	5	4	5
3	3	3	3	3	3	3
5	5	5	5	4	4	1
3	3	4	3	3	3	4
3	4	4	4	4	2	2
4	3	4	3	3	4	2
4	4	4	4	3	2	3
2	3	2	2	3	2	3
4	4	4	2	5	2	3
4	5	4	5	3	3	4
4	2	3	3	3	2	4
4	4	4	5	4	5	4
4	4	4	5	3	2	2
4	3	4	4	3	3	3
4	3	4	4	3	3	2
4	4	4	4	3	3	3
5	3	4	4	3	3	2
4	4	4	4	3	3	3
5	5	5	5	4	4	3
2	4	3	2	4	3	4
3	3	3	3	3	3	2
3	2	3	3	4	3	3
5	5	5	5	3	2	2
4	5	4	4	3	2	1
4	4	4	4	3	3	3
4	4	4	4	5	4	2
5	5	5	5	1	1	1
4	4	3	4	4	3	3
4	3	4	4	5	3	4
5	5	5	5	4	4	4
3	3	4	2	3	5	3
3	3	3	3	2	4	4
4	4	4	4	2	2	3
3	3	2	4	4	4	4
4	5	4	4	5	4	4
4	3	4	4	4	3	4
5	4	5	5	5	4	2
4	4	4	4	4	4	4
3	2	3	4	3	3	4
5	5	4	4	4	3	4
3	4	3	3	3	3	4
3	3	2	2	2	3	3
3	3	3	3	3	3	3
2	3	3	2	3	3	3
2	3	2	2	3	4	2
3	3	4	4	3	2	4
2	3	3	3	2	3	3
4	4	3	2	3	1	1
4	2	3	3	4	3	4
4	5	5	5	2	2	1
4	3	4	4	3	3	4
4	5	5	3	4	3	2
5	5	4	4	2	3	3

4	3	3	4	4	3	2
5	5	5	4	3	1	4
4	4	4	3	5	3	1
4	4	3	4	5	4	3
4	4	4	3	1	2	1
4	3	3	3	3	3	3
3	3	4	4	3	3	3
3	4	4	4	3	4	5
5	5	5	4	4	3	4
3	4	4	4	4	3	4
3	4	4	4	3	3	4
4	4	4	4	2	2	2
4	4	4	4	4	4	4
4	3	2	3	4	5	5
4	5	4	4	2	3	3
4	4	4	4	5	3	3
4	3	3	3	3	4	4
4	5	5	5	5	4	4
4	5	5	5	4	4	5
5	4	4	4	5	5	5
5	4	4	4	5	5	5
5	4	4	4	5	5	5
4	5	4	3	4	5	4
3	4	3	4	4	4	4
4	4	3	4	3	4	4
4	4	3	4	3	3	4
3	3	2	3	3	3	2
4	2	4	5	4	1	4
3	4	4	4	5	4	5
3	2	4	4	3	2	2
3	4	5	4	4	4	3
3	4	3	4	4	5	4
2	5	4	4	3	4	5
3	4	4	4	4	4	4
4	5	4	4	3	4	5
5	4	5	4	5	5	4
5	5	5	5	5	5	5
4	4	4	4	2	3	2
5	5	4	5	5	4	5
5	5	5	5	1	1	5
4	4	4	4	4	3	3
4	4	4	4	4	4	2
5	4	4	4	5	4	4
4	5	5	4	3	3	2
4	3	4	5	3	2	3
4	4	4	4	3	1	2
5	5	5	5	5	5	5
3	4	4	5	4	3	4
3	3	4	2	3	3	2
3	4	4	4	5	4	4
4	2	3	2	1	1	2
4	4	4	4	4	4	4
4	4	4	4	4	3	3
4	4	3	4	3	4	4
4	4	5	4	5	4	5
4	4	5	4	5	4	5

3	1	2	3	5	3	1
3	3	4	3	2	3	2
3	4	4	4	3	2	2
4	3	4	4	2	2	2
3	3	3	3	2	3	2
4	4	4	4	3	3	1
3	3	3	3	3	4	3
3	4	3	3	2	3	3
4	3	4	4	2	3	2
4	4	4	4	2	3	2
4	3	4	4	3	3	2
4	4	4	4	3	3	3
4	4	4	4	3	3	4
4	4	4	4	3	2	1
3	3	2	3	2	3	3
4	4	5	5	4	4	5
4	4	5	3	3	4	4
5	5	5	5	5	5	5
3	4	5	4	5	5	4
4	4	4	4	3	3	1
5	5	5	5	2	2	2
5	5	5	5	3	3	1
4	4	4	4	3	3	3
5	5	5	5	3	2	2
3	3	3	3	2	3	4
3	2	3	3	4	3	2
5	5	5	4	1	1	1
4	4	4	3	3	1	1
5	2	4	1	2	1	2
5	5	5	3	2	3	2
4	4	4	4	2	2	3
5	5	5	4	1	1	1
3	2	4	4	5	3	1
4	3	4	2	2	1	1
4	4	5	4	1	3	1
4	5	4	4	4	5	4
3	3	3	4	4	3	3
5	5	5	5	3	3	3
4	4	5	3	2	2	1
5	2	3	2	1	1	2
3	3	3	3	3	3	3
3	3	4	2	3	5	3
5	5	5	5	2	1	1
5	4	5	5	3	1	1
5	5	5	5	2	2	3
3	3	3	3	2	2	2
5	4	4	4	3	2	3
4	4	4	4	2	2	2
3	3	3	3	3	3	1
4	4	4	4	3	3	3
5	5	4	3	2	2	1
5	4	4	3	3	2	3
5	3	3	4	3	2	1
4	3	4	3	3	3	2
5	3	5	5	4	3	3
4	5	4	4	3	3	3

4	4	4	4	3	3	3
5	4	3	5	3	3	3
4	4	4	4	4	4	4
5	4	4	4	4	4	4
5	5	5	5	3	3	3
4	3	4	3	4	3	4
4	4	4	4	2	3	2
5	5	5	5	3	3	2
5	4	4	5	4	3	4
4	4	4	4	4	4	4
4	3	3	4	3	4	2
4	4	4	5	4	5	4
5	5	3	3	3	3	3
3	3	3	3	3	3	3
3	4	4	4	3	4	4
4	4	4	4	3	2	3
3	3	2	4	4	4	4
3	3	3	3	3	3	3
4	4	4	2	2	3	2
5	4	5	5	5	4	2
4	3	4	4	4	3	4
5	5	5	4	4	3	3
4	3	3	3	4	3	3
5	5	5	5	4	1	1
5	2	5	5	2	5	1
5	5	5	5	1	1	1
4	4	4	4	4	4	4
5	5	5	3	1	2	5
4	3	3	4	5	5	5
5	3	1	2	5	3	2
4	4	2	3	5	3	2
3	5	1	2	5	4	3
3	5	2	1	5	1	4
4	4	4	3	2	2	1
3	4	4	4	4	4	3
2	3	1	1	5	4	1
4	4	3	3	3	2	2
4	3	4	4	5	4	4
5	5	5	5	5	5	5
4	4	4	5	4	4	4
5	5	5	5	5	5	5
4	4	4	4	3	3	3
3	4	4	4	5	4	4
5	5	5	5	5	5	5
5	5	4	5	4	4	4
3	3	3	3	3	3	3
3	4	4	4	2	1	3
4	4	5	5	4	4	5
3	3	4	4	3	2	5
4	3	4	4	3	4	4
4	5	4	4	4	3	3
4	4	4	5	4	5	4
4	5	5	5	4	3	3
4	4	4	4	3	4	4
5	5	5	5	3	3	5
3	3	3	3	3	3	3

5	5	5	5	2	3	3
4	4	4	4	2	2	3
4	4	4	4	1	1	1
3	3	3	3	4	4	4
4	4	5	5	3	4	4
5	5	5	5	3	2	1
4	4	5	5	4	4	5
4	3	5	5	2	2	1
3	4	3	5	4	4	2
4	3	5	5	4	3	3
4	3	4	5	4	3	2
3	3	3	2	4	4	4
4	3	3	4	3	4	3
5	5	5	4	1	1	3
4	3	3	4	4	3	1
3	2	4	2	3	2	4
5	5	4	5	5	4	5
4	4	4	4	2	3	2
5	5	4	5	5	4	5
5	5	5	5	5	5	5
4	4	4	4	3	4	5
4	4	4	4	1	1	1
5	5	5	5	4	4	5
4	4	4	4	3	3	4
4	4	4	4	4	4	4
5	5	5	5	5	5	4
3	3	4	2	3	5	3
3	4	3	3	2	3	3
4	4	4	4	3	3	3
4	4	3	4	4	3	4
3	2	3	3	1	1	2
4	3	4	4	5	3	2
3	4	4	4	3	4	1
5	4	5	5	5	4	5
5	4	5	4	5	5	4
4	5	5	4	4	5	5
3	4	4	4	4	4	4
4	5	5	5	4	3	3
4	3	4	5	3	2	3
4	4	4	4	2	2	2
3	3	4	4	3	3	4
4	3	3	4	3	4	3
4	3	3	2	4	4	2
3	4	4	4	4	4	4
4	4	4	4	3	3	3
5	5	5	5	3	1	3
4	2	2	3	4	2	2
5	4	5	5	3	3	2
2	3	1	1	5	4	1
5	2	4	4	3	3	4
5	5	5	5	5	3	1
4	4	4	4	3	1	3
3	3	3	3	2	2	1
4	4	4	4	3	1	1
5	5	5	5	1	1	1
4	4	4	4	4	4	4

3	4	4	3	3	2	3
5	5	5	5	4	4	4
5	5	5	5	4	2	3
3	5	4	4	3	4	4
5	5	5	4	2	2	2
4	4	4	4	3	4	3
5	4	4	4	3	3	3
3	3	4	3	2	2	3
4	4	4	4	4	4	1
3	4	4	3	3	3	3
4	4	5	4	4	4	4
5	5	5	5	3	2	3
5	5	5	5	3	3	3
5	5	5	5	1	2	3
5	4	5	5	2	1	1
3	4	4	5	3	3	4
3	3	3	3	3	3	3
3	4	5	4	5	5	3
4	4	3	3	2	1	1
4	4	4	4	2	1	1
4	4	4	4	3	4	4
4	3	4	4	4	4	4
4	5	4	4	4	4	5
4	4	4	4	2	2	3
3	3	4	3	4	4	4
4	5	5	5	2	3	3
3	3	3	3	4	2	1
3	4	2	3	4	4	4
4	4	4	4	4	3	3
5	5	5	5	3	3	3
3	3	3	5	4	4	5
3	3	3	5	4	4	5
3	4	4	3	4	4	4
3	3	3	3	1	2	1
4	3	3	4	3	4	4
4	4	4	4	4	4	4
3	3	4	4	4	4	4
1	4	4	3	3	2	2
4	5	4	5	3	3	4
3	4	4	3	3	4	3
3	2	3	2	3	2	3
4	4	4	4	3	3	3
3	4	3	4	4	4	4
4	4	4	3	3	3	3
4	3	4	4	4	5	4
4	4	4	2	2	3	3
3	4	4	3	2	3	3
4	4	4	3	2	3	3
4	4	4	3	3	2	4
5	5	5	1	1	1	1
3	3	4	3	4	3	1
4	4	4	4	2	1	1
5	3	4	5	2	4	3
1	4	4	3	3	2	2
4	3	4	4	3	4	4
3	3	4	4	3	2	1

4	5	4	5	3	3	4
3	4	3	3	3	3	4
3	3	3	3	3	3	3
4	4	3	4	3	3	3
4	4	4	4	5	3	2
3	3	4	2	3	5	3
4	3	4	3	3	3	4
5	5	5	5	4	3	1
3	4	3	2	1	3	4
4	4	4	4	2	2	2
3	4	4	4	3	4	4
4	4	4	4	4	4	5
3	4	4	3	4	3	3
3	4	5	4	3	3	3
3	4	5	3	4	5	3
4	4	4	4	3	3	4
4	4	3	4	2	2	2
4	2	3	3	1	2	3
4	4	4	4	4	5	4
4	4	4	4	4	4	4
4	4	4	4	3	2	3
4	4	4	4	4	4	3
5	5	5	5	4	3	3
4	4	3	3	3	3	4
3	4	4	3	3	2	3
5	4	4	5	3	3	2
5	5	5	5	2	1	1
5	5	5	5	5	4	2
2	4	5	4	3	3	3
2	3	3	4	3	3	3
3	2	3	3	2	3	3
3	3	3	4	4	2	5
3	5	4	2	3	4	4
4	4	4	3	3	4	4
4	4	5	4	3	1	1
4	4	4	4	4	3	3
3	4	4	3	3	4	3
3	4	4	4	4	5	5
4	4	5	5	4	5	4
3	3	4	4	3	3	3
3	4	3	4	3	4	3
3	3	4	4	4	3	3
3	4	3	4	4	4	3
3	4	4	3	4	3	4
3	4	3	4	3	4	3
3	3	1	1	5	3	1
5	4	4	4	4	4	4
4	2	4	4	4	1	4
4	4	3	3	3	3	2
3	4	3	3	3	4	4
4	3	4	4	4	2	2
5	3	4	4	2	2	3
5	5	3	3	2	2	3
3	4	2	2	3	3	3
3	4	3	3	2	3	2
5	3	4	4	2	2	3

4	4	4	4	2	1	2
4	4	5	4	5	3	3
4	4	4	4	2	2	2
4	4	5	4	1	1	1
5	5	5	5	1	1	1
4	4	4	4	1	1	1
3	3	3	3	1	1	1
4	4	4	4	2	2	2
3	3	4	4	5	5	1
2	2	2	2	2	4	5
5	5	5	5	5	5	5
4	4	4	4	4	4	4
4	4	4	4	3	3	4
3	3	4	4	5	5	1
4	3	3	3	4	4	4
5	5	5	5	5	5	5
4	5	4	4	4	3	4
4	3	4	5	2	2	2
4	4	3	3	4	2	2
5	4	5	5	4	2	1
4	4	4	3	4	4	3
4	4	5	3	2	2	1
4	4	4	4	4	3	4
3	3	3	2	4	4	4
4	3	4	5	4	4	4
3	3	3	3	2	2	3
3	3	3	3	3	3	3
3	5	4	4	5	4	5
5	5	5	5	1	2	3
4	5	5	4	5	4	4
3	4	4	4	3	4	4
4	4	3	4	3	4	4
4	4	4	3	3	4	4
4	3	3	3	3	4	2
4	3	4	3	4	3	4
2	3	3	2	3	3	3
4	4	4	4	4	4	3
4	4	4	4	2	2	1
5	5	4	4	2	1	2
5	5	5	5	1	1	1
2	3	3	4	4	4	4
4	4	4	4	4	4	3
3	3	4	4	4	4	3
3	3	3	3	3	3	4
4	3	4	4	2	3	2
5	5	5	5	2	1	5
5	5	5	5	2	2	3
4	4	4	4	2	1	3
4	4	4	4	2	2	2
4	4	4	3	4	3	5
4	4	4	4	3	2	2
4	3	3	3	4	4	3
4	4	4	4	2	2	2
4	4	4	4	4	4	4
3	3	2	4	3	4	5
5	5	5	5	5	5	5

4	4	4	3	2	3	2
3	3	4	4	3	3	3
4	4	4	5	4	5	4
4	5	4	4	2	3	1
4	4	5	4	2	3	1
3	4	3	3	2	2	1
3	4	3	4	2	1	1
5	5	5	5	3	4	3
5	5	4	5	4	4	4
5	4	4	5	4	3	3
2	3	2	2	3	3	2
3	2	3	4	3	3	3
3	2	3	3	3	2	3
2	3	3	3	2	3	3
2	3	2	3	3	3	2
3	3	3	3	5	4	3
5	5	5	5	4	4	4
5	5	5	5	1	2	1
5	5	5	5	4	2	2
4	5	5	5	4	4	4
4	4	4	4	3	4	3
4	4	4	4	3	3	3
4	3	4	4	3	4	3
3	3	4	2	2	3	4
4	4	4	4	4	3	2
4	3	3	4	2	2	3
4	5	3	4	5	4	5
4	3	3	4	3	4	4
4	3	4	2	2	1	1
4	5	4	4	4	4	4
5	5	4	5	3	3	3
5	3	4	5	1	1	1
4	3	4	2	2	1	1
2	3	3	2	2	2	3
3	3	3	3	3	3	2
2	3	2	3	3	2	2
4	3	4	4	3	4	3
4	3	3	4	2	2	3
3	4	4	5	3	4	5
4	3	3	4	2	1	2
3	3	4	3	3	2	3
4	4	4	3	4	2	3
5	3	4	4	4	5	4
4	4	4	3	4	4	4
4	2	4	3	2	3	2
4	3	4	3	2	2	3
4	2	3	3	3	3	2
5	5	5	5	2	3	2
4	3	4	3	3	3	4
4	4	3	3	2	3	3
4	4	4	3	3	3	4
4	4	4	4	4	4	4
4	4	4	4	4	4	4
4	4	4	4	4	2	2
4	5	5	5	4	4	4
4	4	4	4	5	4	4

5	4	5	3	5	4	3
4	4	5	5	5	4	5
5	5	5	4	5	4	4
4	4	4	3	3	3	4
4	4	4	4	4	4	4
5	5	5	5	2	1	4
5	5	4	5	4	5	4
3	3	4	4	3	4	3
3	4	4	4	4	3	4
5	5	5	5	3	3	1
5	5	5	5	3	1	1
3	4	3	3	3	3	4
5	4	5	5	3	2	2
5	4	4	5	3	1	1
5	4	3	3	3	2	2
3	2	3	4	3	2	3
4	3	3	4	2	3	4
4	4	3	3	2	2	2
4	4	5	5	2	2	1
4	5	5	5	4	3	2
3	3	2	4	3	4	5
5	5	4	5	3	3	2
3	3	4	3	4	3	4
4	4	4	4	2	2	2
4	3	4	3	3	3	3
4	4	4	4	3	3	3
3	4	5	5	4	4	3
5	4	4	4	5	4	4
3	3	3	3	2	2	3
5	5	5	5	5	5	5
2	2	2	3	3	3	3
3	3	3	4	4	4	4
4	4	4	5	4	4	5
5	5	5	5	1	1	1
4	4	4	4	1	1	1
4	4	4	4	1	1	1
3	3	3	3	3	3	3
5	5	5	5	5	5	5
5	5	4	5	3	1	1
3	2	3	3	5	3	4
4	4	4	3	3	2	3
5	4	4	4	5	5	5
5	5	5	5	2	2	2
4	4	4	5	2	2	2
5	4	5	5	2	2	2
4	4	5	5	2	2	2
4	3	5	5	2	2	3
2	1	1	2	1	1	2
4	4	5	5	4	4	5
2	1	1	2	1	1	2
4	4	4	4	4	4	4
4	4	5	4	2	2	2
4	4	4	4	4	4	4
3	3	4	2	3	5	3
3	4	3	3	2	2	2
4	4	4	4	2	2	2

4	4	3	4	3	4	3
4	3	4	3	3	4	3
4	4	4	4	2	2	2
4	4	4	4	3	3	4
4	4	4	4	3	3	3
4	4	5	4	4	4	4
4	4	4	4	3	4	4
4	4	4	4	4	4	4
3	3	4	2	3	5	3
4	3	4	4	3	2	1
2	4	5	3	5	4	5
4	5	4	3	2	3	3
3	3	3	4	3	4	3
4	4	4	4	1	1	2
5	4	5	5	3	2	2
3	4	3	3	3	2	4
3	4	3	4	3	1	4
4	3	4	4	4	4	4
3	2	2	4	3	3	5
3	3	4	3	3	2	3
3	3	4	4	5	5	1
4	5	4	4	4	5	5
4	4	4	3	4	4	3
2	1	2	2	2	2	3
2	1	1	2	4	4	4
3	3	4	3	2	2	3
5	4	4	4	3	2	2
3	3	3	3	4	1	1
4	4	4	4	2	3	2
4	4	4	4	2	1	1
3	3	3	3	4	2	2
4	4	4	4	2	1	1
4	4	4	3	5	3	2
3	3	3	3	4	1	1
4	3	3	3	2	1	1
2	1	2	2	2	2	3
2	1	1	2	4	4	4
4	4	4	4	3	3	3
5	5	5	5	1	1	1
5	4	4	4	2	3	3
5	3	4	5	2	2	3
4	3	5	4	2	3	2
4	5	4	5	2	1	1
4	4	4	4	3	3	2
5	5	5	5	3	3	1
4	4	4	4	4	1	4
4	3	3	4	3	4	3
5	1	4	4	3	2	2
5	4	4	4	4	5	4
3	4	5	4	3	4	5
4	4	3	4	3	4	5
2	4	3	5	4	2	3
4	3	3	3	4	4	3
3	3	3	3	4	3	4
4	4	4	4	4	3	3
4	3	3	5	4	4	3

4	3	3	5	4	5	5
4	3	4	5	5	2	3
4	4	4	4	1	2	3
4	5	5	5	3	3	2
5	5	5	5	4	3	3
5	3	4	3	2	1	1
4	5	4	4	4	4	5
4	5	3	3	1	1	2
4	5	4	5	4	4	5
4	3	3	5	2	3	1
5	5	4	3	4	3	3
4	4	4	4	4	4	4
4	5	4	3	4	5	4
3	3	4	3	2	3	2
5	4	4	5	5	4	4
4	5	5	4	4	5	4
5	5	5	5	5	5	3
3	4	3	4	4	3	4
4	4	3	4	4	3	4
3	3	2	3	3	2	2
3	3	3	3	2	3	3
3	3	3	2	2	3	3
3	2	3	3	2	2	3
4	4	4	3	3	2	2
3	3	4	4	3	2	4
5	3	4	5	4	3	4
1	1	1	1	1	1	1
3	3	3	3	3	3	3
4	4	4	4	3	3	3
5	4	5	5	3	4	3
5	4	3	4	4	5	4
4	4	4	4	2	2	2
4	4	3	3	5	5	4
4	4	4	4	4	4	4
4	4	3	3	2	1	1
5	5	5	5	3	2	1
3	4	4	4	2	3	3
5	5	5	5	1	3	1
5	4	4	4	3	3	3
4	4	4	4	3	4	4
4	4	4	4	3	1	1
4	4	3	3	4	4	4
5	5	5	5	4	3	3
5	5	5	5	4	4	2
4	4	4	5	5	5	2
4	4	4	4	4	3	5
4	4	5	5	4	5	4
5	5	5	5	5	4	5
3	3	4	4	2	3	3
4	5	5	5	3	2	4
3	5	4	3	4	4	3
4	4	4	4	4	3	3
5	5	4	5	5	5	4
4	4	4	5	4	4	5
3	4	3	2	3	4	3
4	4	5	5	2	3	1

4	4	4	4	3	1	2
4	3	4	2	2	2	2
5	4	4	4	4	3	3
4	3	4	4	3	2	2
3	4	4	4	2	3	4
4	4	3	3	3	2	2
4	4	5	5	5	4	5
4	4	4	4	2	2	2
4	4	4	4	4	3	3
3	2	3	4	4	3	3
5	5	5	5	2	2	2
2	2	2	3	2	2	2
4	2	4	4	2	2	2
3	3	3	3	3	3	2
3	2	3	3	3	3	2
2	2	2	3	2	2	2
3	2	4	4	3	2	1
4	5	4	3	3	1	2
5	5	5	5	1	1	1
5	4	5	5	3	1	1
5	5	5	5	2	2	2
4	4	4	4	4	4	4
5	5	5	5	3	3	3

DB9	DB10
5	2
2	5
2	3
3	4
4	4
3	5
5	3
3	2
2	3
2	3
4	3
5	5
2	3
2	2
3	4
4	3
3	3
3	3
1	1
3	2
3	3
1	1
3	2
3	1
1	3
1	1
1	1
3	3
2	1
3	3
2	1
4	4
3	3
3	2
4	3
3	4
3	2
2	2
5	5
4	3
2	1
2	2
3	2
3	3
1	1
3	1
1	3
1	2
3	2
2	1
1	1
4	4
4	4
3	1
2	2

4	4
2	1
3	1
4	5
4	4
1	1
4	3
4	4
2	2
2	3
2	2
1	4
2	1
3	2
5	5
2	2
1	1
2	2
3	1
2	2
1	1
3	2
1	1
2	1
3	1
3	3
2	1
3	1
3	2
1	1
4	3
2	2
1	1
3	1
2	3
2	2
3	4
4	4
4	4
5	1
4	4
4	3
5	4
3	3
3	3
3	3
3	3
3	3
4	4
4	4
1	1
3	3
1	1
2	2
4	2
2	2

4	2
3	1
3	1
4	2
2	1
1	1
3	3
4	4
3	3
3	4
3	4
1	1
4	4
4	3
2	2
5	3
5	5
4	4
5	5
5	5
4	4
4	4
4	3
3	4
4	4
3	4
3	4
1	1
4	3
3	4
4	5
4	3
4	3
4	4
4	3
5	4
5	5
4	3
4	4
1	1
3	1
4	4
4	4
1	1
1	2
2	3
5	5
4	5
3	4
3	4
1	4
4	4
3	3
3	3
4	3
4	3

1	2
1	1
1	3
2	2
2	3
3	3
3	4
3	3
3	3
2	3
3	2
3	3
3	3
1	1
4	1
4	5
3	3
5	5
4	4
3	3
2	3
3	3
3	2
3	1
3	3
3	3
1	1
2	1
1	1
1	4
1	3
1	1
1	2
1	1
1	1
4	4
3	4
1	1
2	1
1	4
3	3
3	1
1	3
1	1
1	1
2	2
1	1
3	3
1	1
3	2
1	3
3	4
2	1
1	3
1	1
3	3

3	3
4	4
3	3
3	3
1	4
2	4
2	2
2	3
3	4
4	4
3	4
4	4
3	1
3	3
3	1
3	2
3	4
3	3
2	2
5	1
4	4
4	4
4	5
3	1
3	1
1	1
4	4
1	1
4	4
1	5
1	5
5	4
2	5
1	2
4	3
5	4
2	2
4	4
5	5
5	5
5	5
4	3
3	4
5	5
5	4
4	4
4	3
4	5
4	3
3	4
3	4
3	4
5	3
3	4
3	3
3	3

3	1
1	1
1	1
4	4
4	4
1	1
4	5
2	2
1	1
1	1
3	1
3	1
3	4
2	1
4	3
1	3
4	4
4	3
4	4
5	5
4	3
1	1
5	5
4	4
4	4
5	4
3	1
3	3
4	4
3	4
2	2
4	3
1	2
4	4
5	4
4	3
4	4
2	1
1	2
4	3
3	3
3	4
2	2
4	4
4	4
3	3
4	3
1	1
5	4
1	3
1	1
3	1
1	1
1	1
1	1
4	3

4	2
4	3
2	2
3	4
4	1
4	4
1	3
3	4
5	4
4	4
4	4
3	4
1	3
1	2
1	1
3	2
3	3
3	3
1	1
4	3
3	4
5	4
4	4
2	2
4	4
1	1
1	2
3	3
3	3
2	2
5	3
5	3
3	3
1	1
3	3
4	4
4	4
3	1
3	3
3	4
3	3
3	3
3	3
3	3
3	4
3	3
3	3
3	3
2	1
1	3
5	1
1	1
4	5
3	1
1	1
3	5

1	1
3	3
3	3
4	3
2	2
3	1
5	4
5	3
5	2
1	1
3	3
4	4
2	4
3	4
4	5
3	3
2	2
2	3
5	5
4	4
4	3
4	4
4	3
4	3
3	4
3	2
4	1
1	1
3	4
3	4
3	3
3	5
3	5
4	4
1	1
5	3
4	3
4	4
5	5
4	3
3	4
4	3
3	3
3	4
4	3
1	4
4	4
1	1
3	2
4	4
3	3
1	1
1	1
3	1
3	1
1	1

1	2
4	3
2	2
1	1
1	2
1	1
1	1
2	2
1	1
3	3
5	5
4	4
1	3
1	1
2	3
5	5
4	4
3	2
3	4
1	2
3	1
2	1
4	4
3	1
5	5
3	2
3	3
4	4
4	3
4	4
3	4
3	3
4	4
3	4
3	4
2	3
4	4
1	1
1	1
1	1
5	4
4	3
4	3
3	4
3	2
1	1
2	1
3	3
2	2
2	2
2	1
3	3
2	2
4	4
4	5
5	5

2	2
1	3
5	4
1	2
2	1
1	1
1	1
3	4
5	4
3	4
3	3
4	3
3	3
2	3
2	2
4	3
4	4
1	1
3	1
5	5
4	5
3	3
2	3
4	3
1	4
3	4
4	5
4	4
1	1
3	3
4	3
5	3
1	1
2	3
2	2
2	2
2	1
4	4
4	5
2	1
3	2
1	3
5	4
3	4
1	2
4	3
3	2
1	1
3	3
3	2
5	5
4	4
4	4
2	2
4	4
4	5

4	3
5	5
4	5
3	3
4	4
1	1
5	5
5	4
3	4
2	1
1	1
3	4
1	1
1	2
1	2
2	2
3	4
2	2
2	3
3	2
4	3
4	3
3	4
2	2
3	3
5	3
2	4
5	5
2	2
5	5
3	3
4	4
5	5
1	3
1	2
1	3
3	3
3	3
1	1
4	5
2	2
5	5
2	2
3	2
2	2
2	2
3	2
1	2
5	4
1	2
4	4
3	1
4	4
3	1
2	2
1	3

3	4
2	2
2	2
3	2
4	4
4	5
3	3
4	4
3	1
4	1
1	4
3	3
4	4
1	1
1	1
1	1
1	1
3	3
3	4
2	4
1	1
4	4
3	1
3	1
3	1
3	4
5	2
1	1
1	1
1	1
1	2
1	1
3	1
1	1
1	1
3	1
3	1
3	3
1	1
2	3
3	3
1	1
1	1
2	3
1	2
4	4
3	4
2	2
1	3
4	3
4	3
4	3
4	3
3	3
5	3
2	4

4	5
2	1
3	2
1	1
5	1
1	1
5	5
2	2
4	4
1	1
3	1
4	4
3	4
2	2
4	4
3	4
5	3
3	3
4	4
3	3
3	2
2	2
2	1
1	1
3	2
5	4
1	1
3	3
2	2
2	1
3	4
2	1
4	4
4	4
1	1
1	1
2	2
1	3
1	3
3	4
1	1
4	4
3	4
3	4
3	5
4	4
4	5
4	5
2	2
4	3
3	2
3	3
4	4
4	5
3	2
2	2

1	1
2	2
1	2
2	3
3	1
3	2
5	4
2	2
1	1
3	3
1	1
1	1
4	1
3	3
2	1
3	3
1	1
2	1
1	1
1	1
1	1
4	4
4	1
